# An Unusual Trocar Site Hernia after Prostatectomy

**DOI:** 10.1155/2016/3257824

**Published:** 2016-08-28

**Authors:** Ryan K. Schmocker, Jacob A. Greenberg

**Affiliations:** Department of Surgery, University of Wisconsin School of Medicine and Public Health, Madison, WI, USA

## Abstract

Trocar site hernias are rare complications after laparoscopic surgery but most commonly occur at larger trocar sites placed at the umbilicus. With increased utilization of the laparoscopic approach the incidence of trocar site hernia is increasing. We report a case of a trocar site hernia following an otherwise uncomplicated robotic prostatectomy at a 12 mm right lower quadrant port. The vermiform appendix was incarcerated within the trocar site hernia. Subsequent appendectomy and primary repair of the hernia were performed without complication.

## 1. Introduction

Trocar site hernias are rare complications after laparoscopic surgery with the overall incidence of 0–5.2% [1]. However, with the increasing number of laparoscopic procedures that are performed, these are becoming increasingly common. These hernias are most often associated with ≥10 mm trocars at the umbilical site [1], though there have been reports of trocar site hernias at other locations and with varying port sizes. Here we report an unusual case of a right lower quadrant trocar site hernia following robotic prostatectomy.

## 2. Case

**Figure 1:**
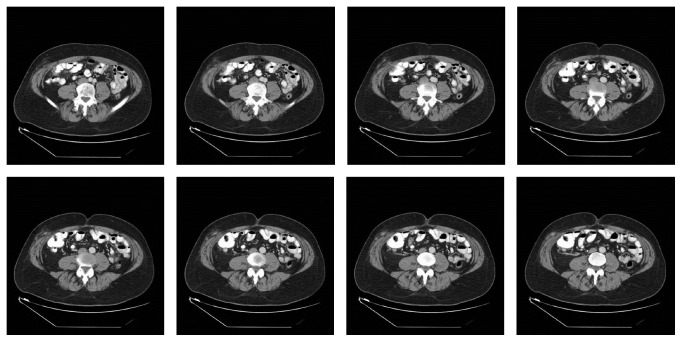
Axial images from a preoperative computed tomography scan demonstrating a right lower quadrant hernia containing fat and the appendix.

A 66-year-old male, with a BMI of approximately 28 kg/m^2^, presented to our surgery clinic for a symptomatic right lower quadrant trocar site hernia, following robotic assisted radical prostatectomy. In December of 2010 he underwent an uncomplicated robotic assisted radical prostatectomy. At the time of the operation a 12 mm trocar was placed in the right lower quadrant, and it is unclear from the operative note whether the fascia was closed at the conclusion of the operation. He recovered from the surgery without issue but unfortunately developed widely metastatic disease over the next 18 months. Upon presentation to our clinic in May of 2012, he was complaining of a two-week history of right lower quadrant pain and a newly discovered mass in the vicinity of a previous trocar site. He initially noted the onset of the pain when performing pull-ups at a local gym, at which time he noted pulling sensation in the right lower quadrant. Following the initial onset of symptoms, he continued to have pain in the area and noted an intermittent bulge as well. He presented for initial evaluation by his primary care provider who obtained a CT scan of the abdomen and pelvis. The scan revealed a 10 mm right lower quadrant hernia with herniated fat and a tubular structure which was read as a possible omental vessel, with concern for incarceration (Figure 1). Upon reviewing the CT with the patient in clinic it was felt that the tubular structure was actually the vermiform appendix and thus laparoscopic incisional hernia repair was recommended.

**Figure 2:**
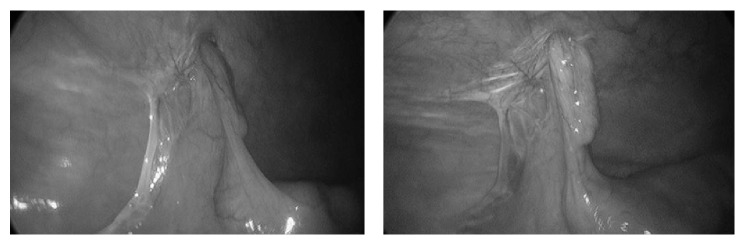
Intraoperative images demonstrating trocar site hernia containing the appendix.

He presented the following week for an elective hernia repair. Pneumoperitoneum was established through a 12 mm umbilical Hasson port. Two additional 5 mm ports were then placed, one in the left lower quadrant and one in the suprapubic area. On inspection of the abdomen there was no evidence of peritoneal disease. The right lower quadrant was then inspected, and as suspected from preoperative imaging a small hernia was identified which contained an incarcerated appendix (Figure 2). The appendix was reduced from the hernia and during the reduction the mesoappendix was injured so a standard laparoscopic appendectomy was performed. Due to the concern for mesh infection following the appendectomy, the hernia was then closed primarily using 0-Ethibond sutures passed transfascially using a suture-passing device. The patient was discharged home from the recovery room and had no postoperative complications at his 2-week follow-up visit. He has no evidence of hernia recurrence at 12 months.

## 3. Discussion

This case illustrates several salient points. While trocar site hernias are relatively rare following laparoscopic surgery, their morbidity can be significant. The relatively narrow aperture of these hernias can result in incarceration requiring an emergent operation and can lead to a bowel resection in rare circumstances. The incidence of overall trocar site hernia varies in the literature from 0 to 5.2% [1], with a recent systematic review showing a pooled incidence of 0.5% [2]. This review found that 82% of trocar site hernias occur at the umbilical port site and 96% of all port site hernias are associated with 10/12 mm trocar sites.

The data on trocar site hernia following robotic prostatectomy in the urology literature is even more limited with the two largest cohorts demonstrating incidences of 0.4% and 0.7% (2/498 and 7/1055), respectively [3, 4]. In these series, similar to the general trocar site literature, the majority of the hernias occurred at 12 mm sites. In the first series, both hernias occurred at the 12 mm umbilical trocar site [3], and in the second 6 of 7 hernias were at the 12 mm right lower quadrant port [4]. The findings in the second series likely reflect the increased use of larger right-sided trocar sites for the assistant's port so that sutures can be passed into the abdomen and handed to the robotic arms.

There have been rare reports of trocar site hernias containing the appendix (Table 1) [5–7]; however to our knowledge this is the first report of an appendix-containing trocar site hernia following robot-assisted laparoscopic prostatectomy. Helgstrand and colleagues reported a trend towards decreased incidence of hernia formation with fascial closure (0.6%) versus no fascial closure (1.5%). Additionally, they also found that increased trocar size was strongly associated with an increased rate of hernia formation [1]. Further, Azurin and colleagues performed a retrospective review of 1300 patients undergoing laparoscopic cholecystectomy and found a rate of trocar site hernia of 0.77%, with all occurring at the 10 mm trocar site [8]. In addition, Erdas and colleagues found that 4.1% of 313 patients following laparoscopic cholecystectomy developed trocar site hernias over a mean follow-up period of 89.8 months [9]. 84.6% of these hernias occurred at the umbilicus while the remainder occurred at the 10 mm subxiphoid port. No trocar site hernias occurred at 5 mm ports or at 10 mm ports that were placed away from the midline.

In conclusion, with an increasing number of laparoscopic cases and therefore trocar site hernias, these hernias could continue to be a source of serious morbidity. Based on our review of the literature, we suggest that routine closure of 10–12 mm trocar sites should be the standard regardless of location, as this could decrease the risk of morbidity from trocar site hernias.

## Figures and Tables

**Table 1:** Previous case reports describing trocar site hernias containing the appendix with subsequent intervention.

Author	Index operation	Trocar size	Onset of symptoms	Hernia contents	Treatment
Goodwin and Ghilchik [5]	Laparoscopic bilateral inguinal hernia repair	Unknown	36 months	Strangulated appendix	Open appendectomy and primary hernia repair
Menenakos et al. [6]	Laparoscopic low anterior resection	12 mm	12 months	Incarcerated appendix	Open appendectomy and primary hernia repair with mesh sublay
Latyf et al. [7]	Laparoscopic anterior resection and partial cystectomy for colovesical fistula	12 mm	18 months	Sliding hernia containing the appendix	Open appendectomy and primary hernia repair with mesh overlay
